# Postpartum follow‐up and Edinburgh Postnatal Depression Scale scores in prenatally suspected placenta accreta spectrum

**DOI:** 10.1002/pmf2.70321

**Published:** 2026-05-21

**Authors:** Alesha White, Ellery R. Cohn, Mishel Malik, Jessica E. Pruszynski, Catherine Y. Spong, Christina L. Herrera

**Affiliations:** ^1^ Department of Obstetrics & Gynecology University of Texas Southwestern Medical Center Dallas Texas USA; ^2^ Parkland Health Dallas Texas USA

**Keywords:** anxiety, EPDS, follow‐up, mental health, placenta accreta spectrum, postpartum depression

## Abstract

**Objective:**

To assess the impact of prenatal suspicion of placenta accreta spectrum (PAS) on attendance of postpartum care and Edinburgh Postnatal Depression Scale (EPDS) scores, with stratification by confirmed PAS based on clinical or pathologic PAS grading.

**Study design:**

Matched cohort of singleton pregnancies that delivered 2010–2024. Patients with prenatal suspicion of PAS were matched to patients without this concern by age (within 5 years), number of prior cesareans, term or preterm delivery, and hypertensive disorders of pregnancy. Exclusions were fetal anomalies, stillbirth, neonatal death, and those without follow‐up data. Maternal demographics, scheduled and attended postpartum follow‐up, and EPDS scores were compared between patients with and without prenatal suspicion of PAS. Sub‐analysis included confirmed diagnosis of PAS (true positive vs. false positive). Availability of EPDS scores was assessed for all cohorts.

**Results:**

Of 202 patients with prenatally suspected PAS, 112 had available EPDS scores. This was different from patients without this concern where 176 scores were available (55% vs. 87%, *p* < 0.001). Although patients with prenatally suspected PAS had high rates of follow‐up at any postpartum visit (87%), they were more likely to be assigned an additional follow‐up visit in our maternal‐fetal medicine (66% vs. 18%, *p* < 0.001), gynecology oncology (14% vs. 0.5%, *p* < 0.001), urogynecology (6% vs. 0.5%, *p* < 0.001) clinics and less likely to attend their routine postpartum follow‐up (59% vs. 88%, *p* < 0.001), and thus have available EPDS scores. Median EPDS scores were not statistically higher comparing those with and without prenatal suspicion of PAS (3 (1–7) vs. 3 (1–7), *p* = 0.971) nor when comparing only patients with confirmed PAS to those without suspicion for PAS (3 (1–7) vs. 3 (1–7), *p* = 0.668) or when comparing patients with clinically or pathologically negative PAS to patients without PAS (3 (1–5) vs. 4 (2–8), *p* = 0.432)). The percentage of EPDS scores that would require intervention and referral was not different among all cohorts.

**Conclusion:**

Patients with prenatal suspicion of PAS were more frequently assigned additional subspeciality follow‐up and less likely to attend their routine postpartum visit and have EPDS scores available. Of patients with prenatally suspected PAS regardless of whether PAS was confirmed, EPDS scores did not differ from patients without this concern. While potentially reassuring, our findings highlight the need for integrated PAS care to address mental health, removing barriers for assessment and enabling referral if indicated.

## 1 INTRODUCTION

Placenta accreta spectrum (PAS) is a highly morbid condition in pregnancy that often requires peripartum hysterectomy [1]. As a traumatic experience, PAS has been associated with higher rates of post‐traumatic stress disorder (PTSD), anxiety, grief, and depression [2, 3]. Existing literature examining the association of mental health disorders with PAS is qualitative. Few studies have used an objective means to demonstrate this association.

Currently, the United States Preventative Services Task Force (USPTF) recommends depression screening in all pregnant and postpartum women with adequate systems in place to provide treatment and indicated follow‐up. The Edinburg Postnatal Depression Scale (EPDS) is a 10‐item questionnaire that is the most commonly used depression screening tool in perinatal care. A prior study using this screening system in patients who underwent an emergent hysterectomy demonstrated increased rates of depression, but similar studies using EPDS have not been completed in patients with a prenatal diagnosis of PAS, regardless of final surgical outcome or pathology [4].

As with many screening tools, the success of depression screening relies on the ability to disseminate the survey. Up to 40% of postpartum patients do not attend their postpartum visit [5]. Data on follow‐up for patients with PAS is limited. We hypothesize that follow‐up for PAS patients is more variable given complicated antenatal and intrapartum courses and thus may be lower. To evaluate depression screening in PAS, where and how these patients follow‐up is a part of the analysis. Therefore, the objective of this study was to assess the impact of prenatal suspicion of PAS on attendance of postpartum care and on postpartum EPDS scores, with stratification by confirmed diagnosis of PAS based on clinical or pathologic grading.

## 2 MATERIALS AND METHODS

This matched cohort study included singleton pregnancies from 2010 to 2024 who had prenatal care, delivery, and postpartum follow‐up scheduled within our system. Patients with prenatal suspicion of PAS based on imaging were matched to patients without prenatal suspicion of PAS based on age (within 5 years), number of prior cesarean deliveries (CD), term or preterm birth (≤37 weeks), and hypertensive disorders of pregnancy. In the case of multiple potential matches, the final match was chosen randomly using R version 4.2.2. Patients with pregnancy complicated by stillbirth after 24 weeks, a major fetal anomaly, neonatal death, or for whom postpartum follow‐up data were unavailable were excluded. This study was approved by our institutional review board.

Patients with a history of CD with an anterior low‐lying placenta or placenta previa are screened for PAS using the placental accreta index (PAI) [6].All who screen positive receive initial counseling in sonography by a maternal‐fetal medicine (MFM) fellow or faculty on the etiology and implications of PAS. If the patient's score has a risk >50%, they are referred to our centralized MFM clinic. Serial PAI is obtained at 28 weeks given potential for trophotrophism. If concern for PAS persists, a magnetic resonance imaging (MRI) scan is completed at 30+ weeks. The MRI provides a topographic description of severity that is used for delivery timing and multidisciplinary planning.

We generally plan delivery between 36 weeks 0 day and 37 weeks 6 days, with earlier delivery individualized based on clinical scenario (e.g., vaginal bleeding). Following discharge after delivery, patients who require hysterectomy are seen in our MFM clinic 1–2 weeks for a post‐operative examination and staple removal if required. All are scheduled for a routine postpartum visit in one of our 11 outlying women's health centers between 2 and 6 weeks postpartum. At this visit, the EPDS questionnaire is administered in English or Spanish. Scores of 13 or greater trigger referral to our behavioral health program for further evaluation.

For this study, maternal and neonatal characteristics between patients with prenatally suspected PAS and patients without this concern were compared. Further sub‐analysis was performed comparing prenatally suspected PAS with confirmation by FIGO clinical or pathologic PAS grading (imaging true positive) to their matches and comparing those where clinical and pathologic diagnosis was negative (imaging false positive) to their match [7, 8]. Maternal characteristics included maternal age; race; rates of chronic hypertension (cHTN), gestational hypertension (gHTN), preeclampsia with severe features (SPE), gestational diabetes mellitus (GDM); number of prior CD; if CD was scheduled or unplanned; gestational age at delivery; maternal length of stay (LOS); rate of postpartum visit follow‐up; and rate of hysterectomy. Neonatal characteristics of interest included rate of neonatal intensive care unit (NICU) admission and infant LOS. These demographics and characteristics were available through an established general obstetrical database and a specific registry for patients with PAS delivered after 2010 at our institution.

Scheduled MFM and routine postpartum visits, visit attendance, and available EPDS scores were extracted by three of the study investigators (A.W., E.C., and M.M.). Median EPDS scores were calculated and compared using the Wilcoxon signed rank test. Rates of postpartum follow‐up and EPDS scores ≥13 were compared using the chi‐square test. Maternal and neonatal demographics were compared using *t*‐tests, Kruskal–Wallis test, chi‐square test, and McNemar's test. All statistical analysis was completed using R‐version 4.2.2.

## 3 RESULTS

**FIGURE 1:**
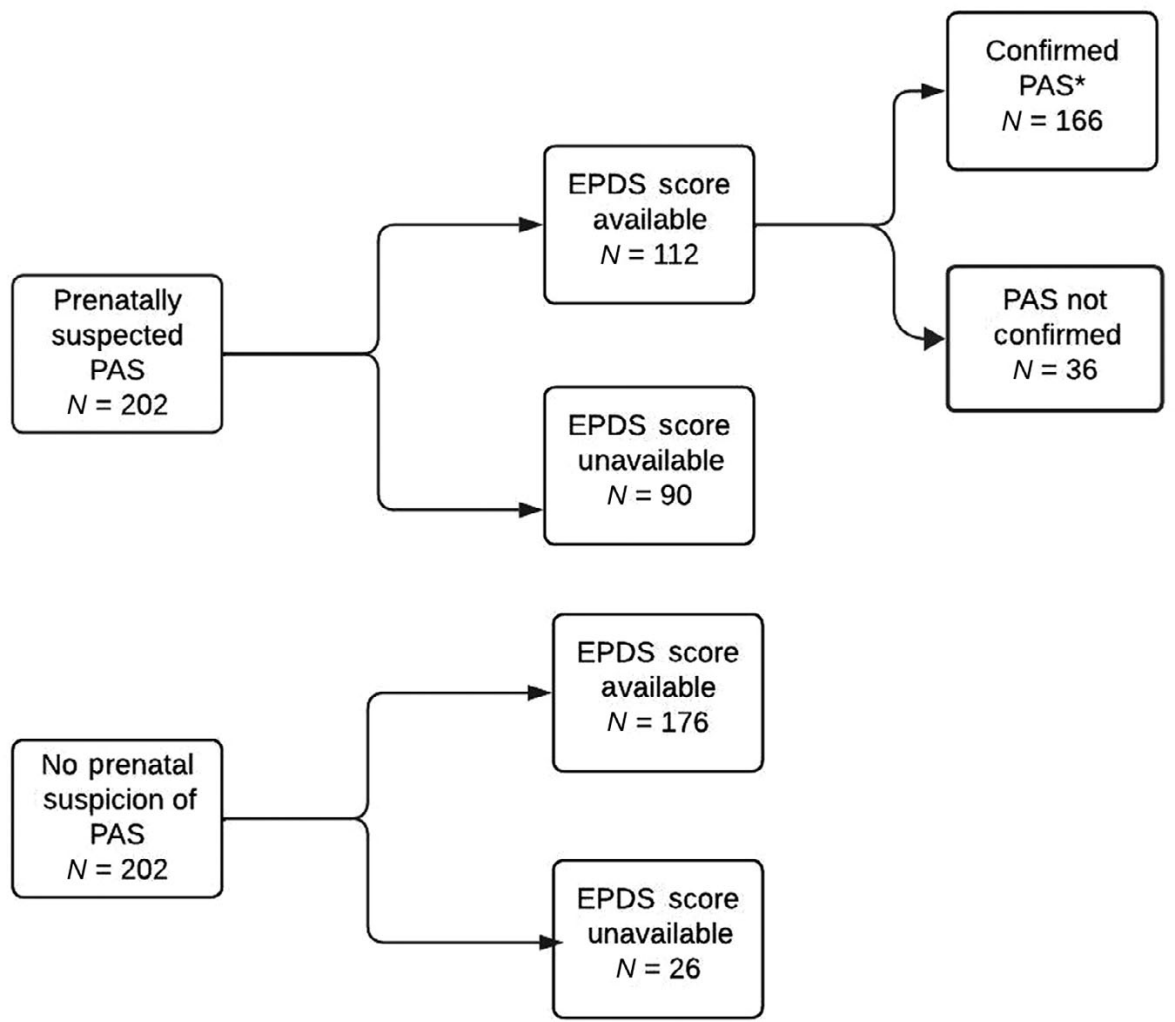
Flow diagram of cases and controls. Note that 202 matches after inclusion and exclusion criteria applied. Of these matches, 112 prenatally suspected PAS patients had available EPDS scores. Based on comparable matches, final 98 matches with EPDS score were compared. *PAS was confirmed either clinically or pathologically.

Note that 236 patients with prenatal suspicion of PAS were matched to patients without this concern. After matching, 34 patients were excluded as they met one or more of the exclusion criteria, leaving 202 matches. Of these matches, EPDS scores were available for 112 (55%) patients with prenatal suspicion of PAS and 176 (87%) patients without this concern (*p* < 0.001). There were 166 (82%) patients with prenatally suspected PAS confirmed based on clinical or pathologic grading (Supplementary Table and Figure 1).

Regarding maternal demographics of all matches with and without EPDS scores, patients with prenatally suspected PAS were more likely to be Black and less likely to be Hispanic when compared to those without this concern (20% vs. 14% and 70% vs. 82% respectively, *p* = 0.043). They were more likely to undergo a scheduled CD (71% vs. 19%, *p* < 0.001) and had a longer median length of stay during their delivery admission (4 (3–5) vs. 3 (3–3) days, *p* < 0.001). There were no additional differences in maternal demographics, rates of NICU admission, or infant length of stay (Table 1).

**TABLE 1:** Maternal and neonatal characteristics based on the presence or absence of prenatal suspicion of PAS.

	Prenatal suspicion for PAS	No prenatal suspicion	
	*N* = 202	*N* = 202	*p*‐value
Age	32.8 (5.3)	32.5 (5.0)	0.213
Race			**0.043**
Black	41 (20)	28 (14)	
White	13 (6)	5 (2)	
Hispanic	142 (70)	165 (82)	
Other	6 (3)	4 (2)	
Number of prior CD	2 (1–3)	2 (1–3)	0.565
Scheduled CD	144 (71)	38 (19)	**<0.001**
cHTN	10 (5)	10 (5)	>0.999
gHTN	20 (10)	20 (10)	>0.999
SPE	14 (7)	14 (7)	>0.999
GDM	27 (13)	29 (14)	0.874
Hysterectomy	160 (79)	2 (1)	**<0.001**
Maternal LOS (days)	4 (3–5)	3 (3–3)	**<0.001**
EPDS scores not available	90 (45)	26 (13)	**<0.001**

Neonatal characteristics			
	*N* = 204	*N* = 213	*p*‐value
GA at delivery	36 (35–36)	36 (34–36)	0.603
NICU admission	72 (35)	61 (29)	0.145
Neonatal LOS (days)	6 (4–12)	5 (4–16)	0.082

*Note*: Data reported as mean (standard deviation), N (%), median (interquartile range).

Abbreviations: CD, cesarean delivery; CHTN, chronic hypertension; EPDS Edinburgh Postnatal Depression Scale; GA gestational age; GDM gestational diabetes mellitus; gHTN, gestational hypertension; LOS length of service; NICU neonatal intensive care unit; PAS, placenta accreta spectrum; SPE, pre‐eclampsia with severe features.

Bolded values are statistically significant.

In terms of postpartum follow‐up, 175 of 202 (87%) patients with prenatally suspected PAS and 190 of 202 (89%) patients without suspected PAS attended at least one postpartum appointment. Patients with prenatally suspected PAS were more likely to be scheduled for a postpartum visit at both MFM clinic and our outlying women's health center (120/202 [59%] vs. 33/202 [16%], *p* < 0.001), but they were less likely to attend both visits (61/120 [51%] vs. 25/33 [76%], *p* = 0.011) and less likely to attend their postpartum visit at the outlying women's health center (110/186 [59%] vs. 175/199 [88%], *p* < 0.001). Thus, they were less likely to have EPDS scores (112/202 [55%] vs. 176/202 [87%], *p* < 0.001). They were also more likely to be scheduled follow‐up with gynecology oncology (29/202 [14%] vs. 1/202 [0.5%], *p* < 0.001) and urogynecology (12/202 [6%] vs. 1/202 [0.5%], *p* < 0.001). Rates of attendance follow‐up in MFM, gynecology oncology, and urogynecology visits did not differ. Similar patterns of follow‐up were observed when PAS was prenatally suspected and confirmed with 89% (147/166) of these patients attending at least one postpartum appointment; however, when clinical or pathologic diagnosis was negative, rates of MFM, outlying women's health center visits, gynecology oncology and urogynecology visits, and attendance did not differ (Table 2).

**TABLE 2:** Postpartum follow‐up and availability of Edinburgh Postnatal Depression Scale (EPDS) scores based on presence or absence of prenatal suspicion for placenta accreta spectrum (PAS).

Prenatally suspected PAS vs. no prenatal suspicion			
	Prenatally suspected PAS	No prenatal suspicion	
	*N* = 202	*N* = 202	*p*‐value
EPDS available	112 (55)	176 (87)	**<0.001**
Median EPDS score^a^	3 (1–7)	3 (1–7)	0.971
EPDS ≥13^a^	8 (8)	3 (3)	0.248
Scheduled for both MFM and WHC	120 (59)	33 (16)	**<0.001**
Attended both^b^	61 (51)	25 (76)	**0.011**
EPDS available^c^	60 (98)	25 (100)	0.52
Scheduled for MFM	133 (66)	36 (18)	**<0.001**
Attended MFM^b^	122 (92)	32 (89)	0.595
EPDS available^c^	63 (52)	25 (78)	**0.007**
Scheduled for WHC	186 (92)	199 (99)	**0.002**
Attended WHC^b^	110 (59)	175 (88)	**<0.001**
EPDS available^c^	108 (98)	175 (100)	0.073
Scheduled for gynecology oncology	29 (14)	1 (0.5)	**<0.001**
Attended gynecology oncology	25 (86)	1 (100)	0.69
EPDS available	11 (44)	1 (100)	0.271
Scheduled for urogynecology	12 (6)	1 (0.5)	**0.002**
Attended urogynecology	12 (100)	0 (0)	**<0.001**
EPDS available	7 (58)		

Prenatally suspected and confirmed PAS vs. no prenatal suspicion			
	Confirmed PAS	No prenatal suspicion	
	*N* = 166	*N* = 166	*p*‐value
EPDS available	84 (51)	146 (88)	**<0.001**
Median EPDS score	3 (1–7)	3 (1–7)	0.668
EPDS ≥ 13	7 (9)	2 (3)	0.405
Scheduled for both MFM and WHC	112 (67)	26 (16)	**<0.001**
Attended both^b^	55 (49)	20 (77)	**0.01**
EPDS available^c^	54 (98)	20 (100)	0.544
Scheduled for MFM	125 (75)	29 (17)	**<0.001**
Attended MFM^b^	115 (92)	25 (86)	0.328
EPDS available^c^	57 (50)	20 (80)	**0.006**
Scheduled for WHC	150 (90)	163 (98)	**0.002**
Attended WHC^b^	83 (55)	145 (89)	**<0.001**
EPDS available^c^	81 (98)	145 (100)	0.06
Scheduled for gynecology oncology	28 (17)	1 (1)	**<0.001**
Attended gynecology oncology	24 (86)	1 (100)	0.684
EPDS available	10 (42)	1 (100)	0.25
Scheduled for urogynecology	12 (7)	1 (1)	**0.002**
Attended urogynecology	12 (100)	0 (0)	**<0.001**
EPDS available	7 (58)	–	–

Prenatally suspected but negative for PAS vs. no prenatal suspicion			
	Prenatally suspected but negative for PAS	No prenatal suspicion	
	*N* = 36	*N* = 36	*p*‐value
EPDS available	28 (78)	30 (83)	0.773
Median EPDS score	3 (1–5)	4 (2–8)	0.432
EPDS ≥ 13	1 (4)	1 (4)	0.312
Scheduled for both MFM and WHC	8 (22)	7 (19)	0.772
Attended both^b^	6 (75)	5 (71)	0.876
EPDS available^c^	6 (100)	5 (100)	–
Scheduled for MFM	8 (22)	7 (19)	0.772
Attended MFM^b^	7 (88)	7 (100)	0.333
EPDS available^c^	6 (86)	5 (71)	0.515
Scheduled for WHC	36 (100)	36 (100)	–
Attended WHC^b^	27 (75)	30 (83)	0.384
EPDS available^c^	28 (100)	30 (100)	–
Scheduled for gynecology oncology	1 (3)	0 (0)	0.314
Attended gynecology oncology	1 (100)	—	–
EPDS available	1 (100)	—	–
Scheduled for urogynecology	0 (0)	0 (0)	–
Attended urogynecology	—	—	–
EPDS available	—	—	–

Abbreviations: EPDS Edinburgh Postnatal Depression Scale; MFM, maternal fetal medicine; SICU, surgical intensive care unit; WHC, women's health center.

^a^ Represents all available EPDS scores for all cases and controls.

^b^ Denominator (*n*) for percentage is the number of scheduled patients above within same section.

^c^ Denominator (*n*) for percentage is the number of patients that attended that particular visit within the same section.

If EPDS scores were available, the median EPDS score for patients with prenatally suspected PAS was not different from patients without this concern (3 (1–7) vs. 3 (1–7), *p* = 0.971). The rate of EPDS scores of ≥13 did not differ (8% vs. 3%, *p* = 0.248). EPDS scores further did not differ when comparing patients with prenatally suspected and confirmed PAS to patients without this concern (3(1–7) vs. 3 (1–7), *p* = 0.668) or when comparing patients with prenatally suspected PAS but negative at delivery or on pathology to patients without this concern (3 (1–5) vs. 4 (2–8), *p* = 0.432). The rate of EPDS scores ≥13 did not differ regardless of whether PAS was confirmed at delivery (Table 2).

## 4 CONCLUSION

We observed a high rate of postpartum follow‐up in all patients included in this analysis at rates higher than the national average. While patients with prenatal diagnosis of PAS attended postpartum follow‐up at high rates, they were more likely to be scheduled for and attend additional subspecialty follow‐up and less likely to attend their routine postpartum visit. Since the routine postpartum visit at our outlying women's health center is where patients are screened with EPDS, they were less likely to undergo screening. This remained true for patients with prenatally suspected and confirmed PAS.

The lack of follow‐up at our outlying women's clinic noted in our PAS population is likely due to the multiple appointments that are part of our system of postpartum care. Patients who undergo hysterectomy for PAS are seen in our MFM clinic soon after delivery for a postoperative exam but an EPDS questionnaire is not administered. It is not until the patient follows up in the outlying women's health center that they are administered this questionnaire. Patients may not follow up at this visit if they are seen initially in the MFM clinic because they are unaware or do not see the utility of the additional appointment. Our findings implicate that mental health screening should be consistently integrated into the care of PAS patients with complex treatment and follow‐up to fully assess the impact. Specifically for our patient population, we acknowledge that a practice change is needed such that EPDS screening should take place at our MFM clinic to fully capture the patient experience. We assume that lack of difference observed is biased and heavily influenced by decreased follow‐up rates and missing data.

In conclusion, patients with prenatally suspected and confirmed PAS attended postpartum follow‐up at rates higher than nationally reported averages; however, they are more likely to have multiple postpartum visits and less likely to attend routine postpartum and be screened for postpartum depression. Patients with prenatally suspected and confirmed PAS should be offered integrated care that addresses mental health, removing barriers for assessment and enabling referral if indicated. Supporting Information

## AUTHOR CONTRIBUTIONS

**Alesha White**: Conceptualization; writing—original draft; writing—review and editing; investigation; methodology; data curation. **Ellery R. Cohn**: Writing—review and editing; investigation; data curation. **Mishel Malik**: Writing—review and editing; investigation; data curation. **Jessica E. Pruszynski**: Formal analysis; data curation; writing—review and editing. **Catherine Y. Spong**: Conceptualization; writing—review and editing. **Christina L. Herrera**: Conceptualization; investigation; writing—review and editing; methodology; supervision.

## CONFLICT OF INTEREST STATEMENT

The authors declare no conflicts of interest.

## Supporting information




**Supplementary Table**. Clinical grade and pathology of patients with prenatally suspected placenta accreta spectrum
