# Supplementary material for: Postpartum follow‐up and Edinburgh Postnatal Depression Scale scores in prenatally suspected placenta accreta spectrum
Source: Pregnancy (Hoboken). 2026 May 21;2(3):e70321. doi: 10.1002/pmf2.70321 (PMC13344216; doi:10.1002/pmf2.70321)
Supplement: Supplementary file 1 [file PMF2-2-e70321-s001.docx]

**Supplementary Table.** Clinical grade and pathology of patients with prenatally suspected placenta accreta spectrum

| **FIGO Clinical Grade^7^** | |
| --- | --- |
| **Grade 1**  **Grade 2**  **Grade 3**  **Grade 3A**  **Grade 3B**  **Grade 3C**  **PAS but not specified**  **Negative**  **Not described** | 17 (8)  103 (51)  2 (1)  12 (6)  10 (5)  5 (2)  14 (7)  33 (16)  6 (3) |
| **Pathology as per the Society of Pediatric Pathology^8^** | |
| **BPMF**  **Grade 1**  **Grade 2**  **Grade 3A**  **Grade 3D**  **Grade 3E**  **Negative**  **Ungradable**  **No pathology**  **Not described** | 3 (1)  12 (5)  13 (6)  81 (40)  22 (11)  13 (6)  42 (21)  13 (6)  1 (0.4)  2 (1) |
| **Data reported as n (%).**  ***BPMF* basal plate myometrial fibers; *PAS* placenta accreta spectrum.** | |
